# Biochemical Battle: Influence of Omega-6 Fatty Acids on the Formation of DNA Adducts with 4-HNE

**DOI:** 10.3390/cimb47080645

**Published:** 2025-08-12

**Authors:** Edyta Błaszczyk, Bolesław T. Karwowski

**Affiliations:** Food Science Department, Faculty of Pharmacy, Medical University of Lodz, ul. Muszyńskiego 1, 90-151 Lodz, Poland; edyta.blaszczyk@umed.lodz.pl

**Keywords:** lipid peroxidation products, 4-hydroxynonenal, polyunsaturated fatty acids, 4-HNE adducts, DNA damage

## Abstract

While omega-6 fatty acids play an important role in normal cell function, their excess in the diet is associated with an increased risk of developing diseases such as obesity, non-alcoholic fatty liver disease (NAFLD), inflammatory bowel disease (IBD) and Alzheimer’s disease. Furthermore, excessive intake has been shown to lead to chronic inflammation, which is related to increased production of reactive oxygen species (ROS). This conditioncan initiate lipid peroxidation in cell membranes, leading to the degradation of their fatty acids. One of the main products of omega-6 peroxidation is the α,β-unsaturated aldehyde, i.e., 4-hydroxynonenal (4-HNE), which is able to form four diastereoisomeric adducts with guanine. These 4-HNE adducts have been identified in the DNA of humans and rodents. Depending on their stereochemistry, they are able to influence double helix stability and cause DNA–DNA or DNA–Protein cross-links. Moreover, studies have shown that 4-HNE adducts formed in the human genome are considered mutation hotspots in hepatocellular carcinoma. Although the cell possesses defence mechanisms, without a well-balanced diet allowing correct cell function, they may not be sufficient to protect the genetic code. This review provides an overview of the molecular mechanisms underlying oxidative stress, lipid peroxidation, and the formation of DNA adducts. Particular emphasis is placed on the role of an omega-6-rich diet in inflammatory diseases, and on the formation of 4-HNE, which is a major product of lipid peroxidation, and its broader implications for genome stability, ageing, and disease progression.

## 1. Introduction

**Figure 1:**
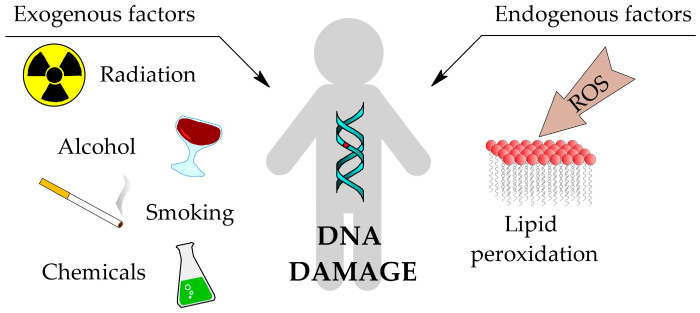
Common sources of DNA damage [1].

All cells in the human body are subject to the processes of ageing, mutations and finally carcinogenesis. These processes can be hastened by interactions between various exogenous and endogenous factors with the cell, which influence the excessive production of reactive oxygen species (ROS). Exogenous factors primarily include radiation (ionising and non-ionising), chemicals, smoking and excessive alcohol consumption [1]. ROS are natural byproducts of cell metabolism, therefore their endogenous source is mitochondria. However, they can also be produced in the endoplasmic reticulum, lysozymes and peroximes, where oxidation processes occur naturally [2]. ROS can lead to oxidative damage of proteins, fatty acids and DNA present in the cell. This causes structural changes in these biomolecules and as a result, they may not be able to perform their proper functions, or their ability may change (Figure 1) [3].

Fatty acids are the main building blocks of cell membranes (lipid bilayer) [4]. Thus, they are exposed to ROS from both the outside and inside of the cell. ROS initiate peroxidation of fatty acids, leading to their degradation and the formation of reactive compounds, i.e., aldehydes. The degradation products depend on the type of the fatty acid. In the case of omega-6 (ω-6), one of the main products is the α-β unsaturated aldehyde—4-hydroxynonenal (4-HNE) [5]. Therefore, 4-HNE is an important biomarker of lipid peroxidation [6].

The ω-6 fatty acids are important in the human diet due the role they play as building blocks of the cell membrane and factors contributing to the inflammatory response. However, ω-6 and ω-3 fatty acids compete with each other for inclusion in cell membranes and for enzyme active sites. Therefore, a diet high in ω-6 leads to elevated levels of its metabolites, that increase inflammatory processes, during which ROS are produced [7].

The aim of this article is to highlight the effect of excessive intake of ω-6 fatty acids on the formation of DNA adducts, as well as to describe the mechanisms that lead to their formation and the resultant genotoxicity.

## 2. Omega-6 Fatty Acids—Friend or Enemy?

Essential fatty acids (EFAs) are polyunsaturated fatty acids necessary for proper bodily function and health. As the human body cannot synthesise them, they must be provided with food.

**Figure 2:**
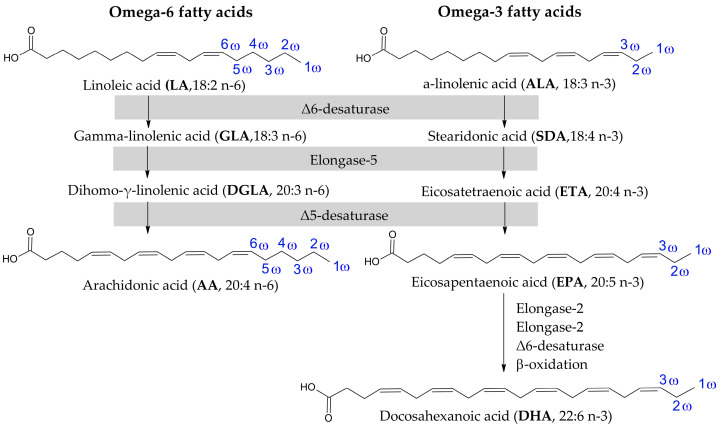
Structures of representatives of ω-6 and ω-3 fatty acids [7].

EFAs are divided into two families, i.e., ω-3 and ω-6, categorised by the position of the first double bond in the chain from the methyl (ω) end (Figure 2). Two well-known representatives of ω-6 are linoleic acid (LA; 18:2 n-6) and arachidonic acid (AA; 20:4 n-6). LA is derived mainly from vegetable oils, such as safflower, sunflower, soybean and corn oils. Significant amounts are also present in nuts [7] and highly processed foods [8]. It is one of the building blocks of the mitochondrial membrane [9]. In mammalian cells, LA is enzymatically converted into AA by elongation of the carbon chain and the formation of two more double bonds [7]. The source of AA in food is mainly fatty meats, including pork, duck and turkey [10]. It is also found in milk and eggs, although levels can vary depending on the animal’s diet [11,12]. Additionally, in the cell, it occurs as a key component of cell membranes, where it affects their fluidity and functionality [13].

On the other hand, the ω-3 group includes alpha-linolenic acid (ALA; 18:3 n-3), the substrate, converted by the same enzymes as in the case of ω-6, and forms another two acids in this family, i.e., eicosapentaenoic acid (EPA; 20:5 n-3) and docosahexaenoic acid (DHA; 22:6 n-3) (Figure 2). All ω-3 are mainly obtained from oily fish and algae [7].

AA and EPA are primarily substrates in the synthesis of tissue hormones, eicosanoids. The process for both substrates requires the same enzymes, resulting in the competition of their active sites.

**Figure 3:**
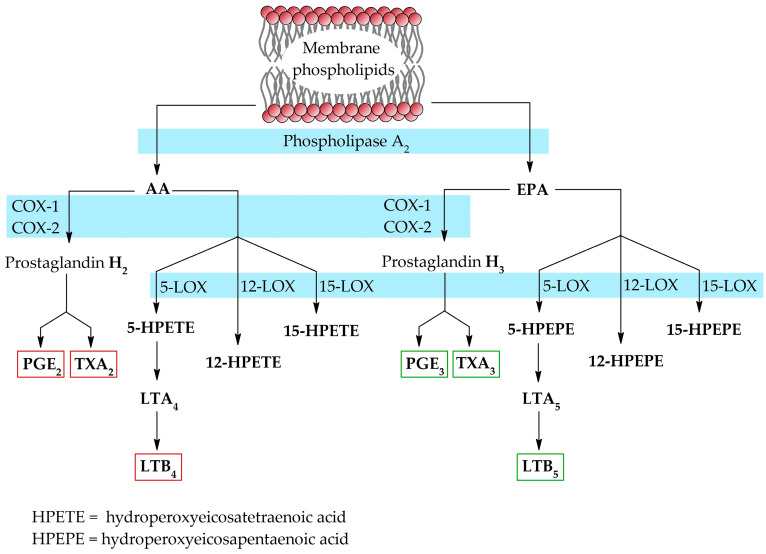
Biosynthesis of eicosanoids from EPA and AA [7,14].

The synthesis of eicosanoids begins with the hydrolysis of fatty acids from cell membranes by phospholipase A2. Then, the released fatty acid can be converted to prostaglandins (PGs) by cyclooxygenase enzymes (COX) and further to thromboxanes (TXs), or to the corresponding hydroperoxides (HPETEs or HPEPEs) by lipooxygenase enzymes (LOX). However, only isomers of fatty acids with a hydroperoxide group at position C5 are further converted to leukotrienes (LTs). The AA-derived eicosanoids, including PGE2, TXA2 and LTB4, are pro-inflammatory mediators. In contrast, EPA-derived eicosanoids, i.e., PGE3, TXA3 and LTB5, are anti-inflammatory [14] (Figure 3).

Since the human body is unable to convert ω-6 to ω-3, it is crucial to maintain the recommended optimal dietary ratio (ω-3/ω-6 1:2). However, the Western diet is currently dominated by highly processed foods, high meat consumption and a relatively low intake of fish, vegetables and seeds. It all has led to a significantly higher ratio between ω-3 and ω-6 fatty acids, which is now estimated to be 1:15 [7]. This imbalance has been reflected in the increased prevalence of civilisation diseases, including obesity [15], non-alcoholic fatty liver disease (NAFLD), inflammatory bowel disease (IBD) and even Alzheimer’s disease (AD) [16].

These diseases are correlated with a diet rich in omega-6. Some experimental and observational studies have examined the effect of this diet on various models (Table 1). Studies on rodents have shown increased lipogenesis and lipid accumulation in the liver, along with elevated levels of proinflammatory cytokines such as TNF-α and IL-6. This results in body-weight gain [16,17,18,19], hepatic steatosis [17,18,19,20], insulin resistance [17,18,20,21] and changes in their gut microbiota [21], all of which are linked to obesity and NAFLD. Additionally, such a diet causes gut dysbiosis [22,23,24], increasing the proliferation of pro-inflammatory microbes [24] and exacerbating colonic inflammation [24,25]. These factors have been shown to promote intestinal damage in colitis models [26,27], providing a potential link with IBD, such as ulcerative colitis.

In transgenic mouse models of Alzheimer’s disease, elevated ω-6 intake has been shown to promote the deposition of amyloid-β [28,29] and disrupt synaptic integrity [30], suggesting a potential involvement in neurodegenerative processes.

These studies also reported an altered lipid profile in the plasma, gut, liver and brain, characterised by an increased proportion of ω-6.

Observational studies on humans suggest that high dietary ω-6 intake is associated with an increased risk of weight gain [31] and developing ulcerative colitis [32,33], although more longitudinal and interventional studies are needed to establish causality.

**Table 1:** Adverse effects of a diet rich in ω-6.

Associated Diseases	Experimental Model	Metabolic and Inflammatory Outcomes	Reference
Obesity, Non-alcoholic fatty liver disease	Rats	Body-weight gain, hepatic steatosis, insulin resistance, elevated levels of pro-inflammatory cytokines, changes in gut microbiota	[17,21]
	Mice	Body-weight gain, increased lipogenesis, hepatic steatosis, insulin resistance	[18,19,20]
	Human	Body-weight gain	[31]
Inflammatory bowel disease, Ulcerative colitis	Rats	Acute inflammatory changes in the colonic structure	[27]
	Human	Increased risk of ulcerative colitis	[32,33]
	Mice	Dysbiosis, increased colitis severity, intestinal damage, proliferation of pathobionts	[22,23,24,25,26]
Alzheimer’s disease	Mice	Promotes the deposition of amyloid-β, disruption of synaptic structures	[28,29,30]

## 3. Pathways of Formation of 4-Hydroxynonenal

The mechanism of 4-HNE formation has been a matter of debate for many years. The initial observations were made by Esterbauer et al. The results of their studies, with the use of microsomes and Fe^2+^ salt, indicated that this process involved the formation of fatty acid peroxides and their subsequent degradation [34].

**Figure 4:**
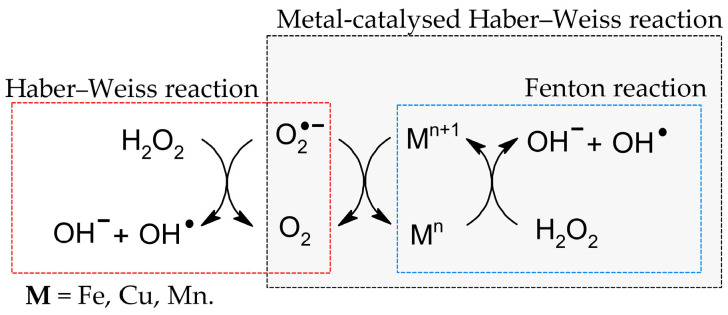
Fenton and Haber–Weiss reactions with and without metal catalysis [39,40].

As it is known today, the formation of fatty acid peroxides is primarily initiated by ROS during oxidative stress. However, it must be remembered that fatty acid peroxides can also be formed enzymatically by LOX and COX [35,36]. In the cell, the main source of ROS is the mitochondria, where H_2_O_2_ is formed as an intermediate of oxygen metabolism. This is a low-reactive molecule which can diffuse across cell membranes [37]. Nevertheless, an increase in the concentration of transition metal ions, i.e., Fe^2+^, Cu^2+^ and Mn^2+^ in the presence of H_2_O_2_ [38], leads to the Fenton [39] and Haber–Weiss reactions with and without metal catalysis [40], which results in the formation of excessive amounts of the hydroxyl radical (OH^●^) (Figure 4) [41].

This small and highly reactive molecule begins the process of lipid peroxidation, a chain of reactions that lead to the degradation of lipids. The initial step in this process is the extraction of a hydrogen atom (hydrogen abstraction) from the bis-allyl position of a fatty acid by OH^●^.

The resulting radical is then rearranged into a more stable, resonance-stabilised radical to which oxygen is added to form the peroxy radical (LOO^●^). Thus, the propagation step of lipid peroxidation starts. Subsequently, reinitiation of peroxidation can occur by the extraction of a hydrogen atom by LOO^●^ from the next fatty acid. It leads to the production of 9/13-hydroperoxyoctadecadienoates (9/13-HPODEs) from linoleic acid, and 11/15-hydroperoxyeicosatetraenoate (11/15-HPETE) from arachidonic acid [42].

**Figure 5:**
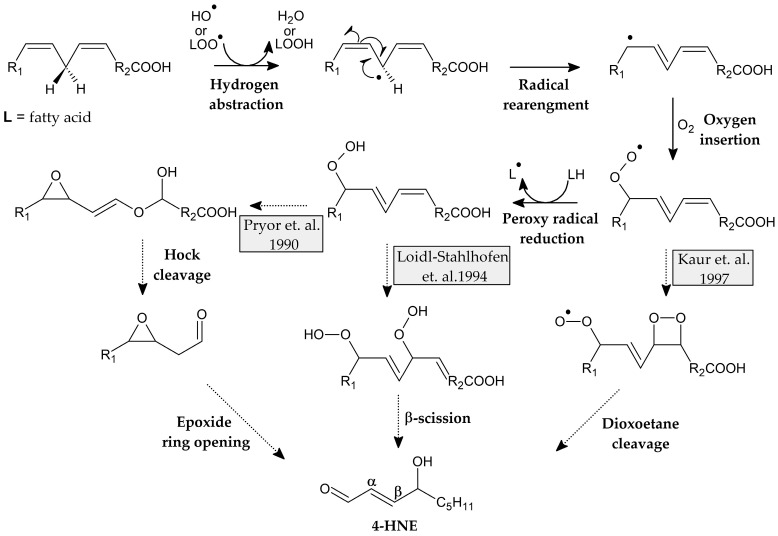
Proposed mechanisms of formation of fatty acid peroxides and structures of intermediate products [43,44,45].

However, the degradation of these peroxides is still under discussion. Pryor et al. suggest that 4,5-epoxyhydroperoxide may serve as an intermediate that undergoes Hock rearrangement and degradation [43]. A more recent study of linoleic acid autoxidation by Loidl-Stahlhofen et al. suggests the formation of dioxygenated products that undergo β-scission [44], whereas Kaur et al. postulate that a dioxoethane derivative may be formed as an intermediate, which then undergoes further degradation in the oxidation of Low-Density Lipoproteins (LDL) (Figure 5) [45].

**Figure 6:**
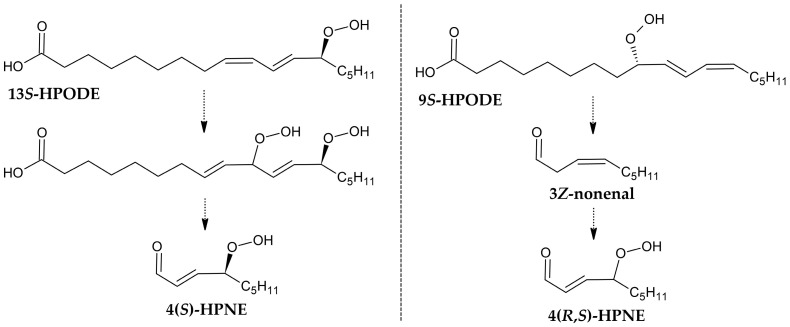
Two proposed routes, based on the chirality analysis of the products [47,48].

Schneider et al. proposed two routes for the formation of 4-hydroxyperoxynonenal (4-HPNE) (Figure 6). The first involves the decomposition of 13-HPODE, which does not alter the chirality of the product 4-HPNE [46]. However, in the case of the second, i.e., from 9*S*-HPODE, a racemic mixture is formed instead [47], which suggests the formation of a 3*Z*-nonenal by a non-enzymatic pathway, as noted in the plant cell [48].

**Figure 7:**
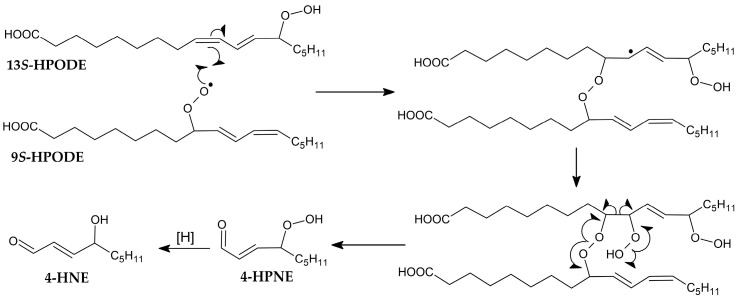
Proposed mechanism of intramolecular peroxyl addition [50].

However, this theory was later extended with a further mechanism through which the peroxyl radical dimerises with another fatty acid molecule, thus allowing intermolecular oxygen transfer [49]. Based on further research on cardiolipin oxidation, a more distinct mechanism was proposed involving the addition of an intramolecular peroxyl radical and decomposition (Figure 7) [50].

## 4. DNA Adducts Formation and Their Properties

**Figure 8:**
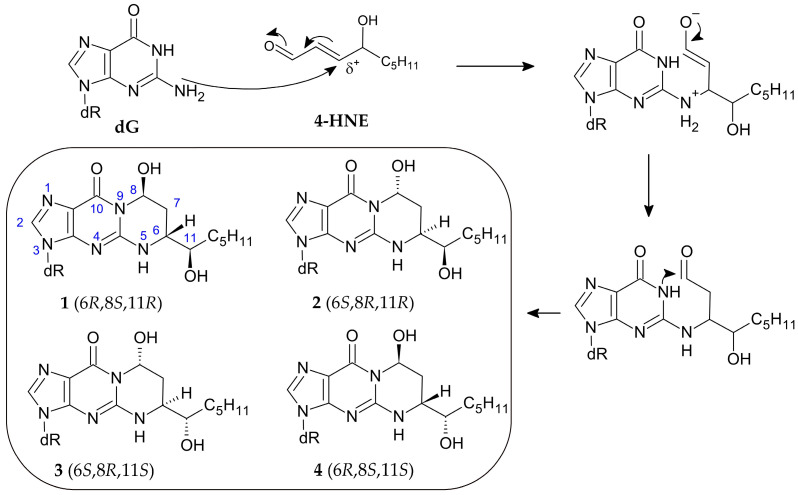
Mechanism of 1,*N*^2^-propano-2′-deoxyguanosine adduct formation and its four diastereoisomers [53,55].

Due to the electrophilic nature of the β position, 4-HNE reacts readily with nucleophilic molecules to form adducts. The reactivity of the aldehyde varies, depending on the substrate, in the following order: dG > dC > dA > dT [51]. When reacting with the genome, 4-HNE forms 1,*N*^2^-propano-2′-deoxyguanosine adducts as the main product rather than multiple products with various nucleic bases [52]. The exocyclic ring is formed by Michael addition to the N^2^ exocyclic amine group, followed by cyclisation to the N^1^ nitrogen. Interestingly, the reaction with dG leads to the formation of four cyclic diastereoisomers under physiological conditions [53] but only compounds **1** and **3** as a result of the reaction with calf thymus DNA [54] (Figure 8).

Studies have shown that these rather bulky adducts influence DNA stability, and this is an isomer-dependent matter (Table 2). Oligonucleotides containing adducts **1**, **2**, **3**, and **4** were obtained by stereospecific synthesis, and they were used to form the corresponding duplexes. The stability of the created duplexes was investigated by measuring the melting temperatures (T_m_), which were associated with hydrogen bonding between the base pairs and base stacking interactions [56]. In general, all duplexes containing those adducts destabilised the structure, which resulted in a lower T_m_ compared to the unmodified duplex, for which T_m_ was 65 °C. The most destabilising effect was shown by isomer **3**, whose T_m_ was 49 °C, followed by isomer **1** (T_m_ = 53 °C). Additionally, the same isomer **3** was found to be able to form an interstrand cross-link in a CpG context [55].

Due to their opposite configurations, stereoisomers **1** and **3** merited special attention. Formation of a duplex which contains adduct **3** and the complementary dC resulted in the opening of an exocyclic ring, allowing the free aldehyde group (**5**) to react with the nearest amino group by nucleophilic addition. This created carbinolamine cross-link **6,** which can undergo dehydration to the imine-type cross-link **7**. These reactions were reversible and occurred quite slowly.

**Figure 9:**
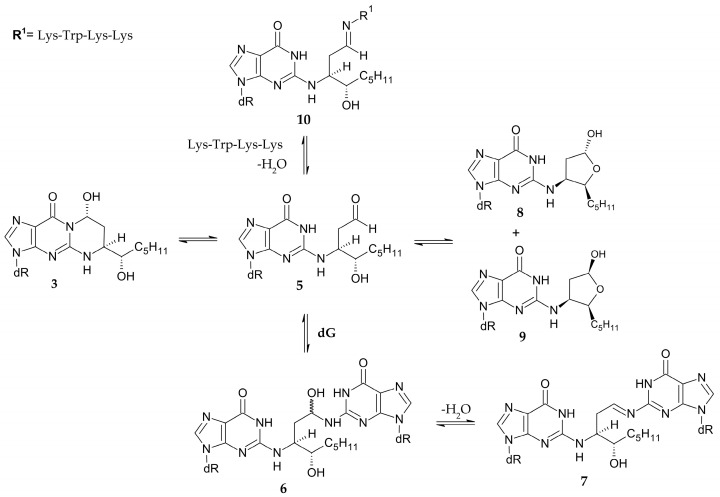
Formation of cross-links and hemiacetals by 1,*N*^2^-propano-2′-deoxyguanosine adduct [55,57,58].

However, the yield was high, i.e., 85% in 61 days. It was also observed that the formed aldehyde was in equilibrium with the cyclic hemiacetals (**8**, **9**), which could be the reason for the low rate of cross-link formation [57]. Moreover, the duplex containing the same isomer **3** formed a cross-link with the Lys-Trp-Lys-Lys tetrapeptide (**10**) much faster than the other adducts (Figure 9) [58].

It has been noted that all of these adducts can be found in human cells. Adduct accumulation, quantified using the ^32^P-post labelling method, was found to be similar in brain neurons in Alzheimer’s patients and control subjects of the same age [59,60]. Moreover, 4-HNE has also been found to readily form adducts at codon 249 of the human *p53* gene, which is considered a mutational hotspot in hepatocellular carcinoma, with G → T transversion occurring in more than 50% of studied cases [61]. When conducting studies on the mutagenicity of the isomers, Fernandes et al. found that two stereoisomers, **1** and **3**, induced four times more G → T transversion mutations in mammalian cells [62]. Therefore, there appears to exist a relationship between diet and DNA damage, which may play a role in various diseases.

## 5. Defence Mechanisms

**Figure 10:**
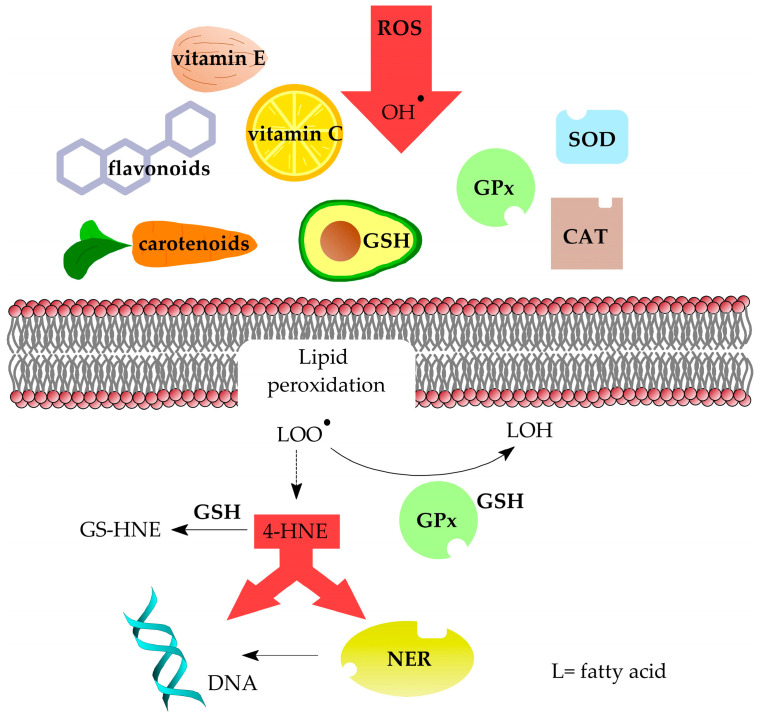
Ways in which cells are protected against oxidative stress, lipid peroxidation products and formation of DNA damage.

To reduce the effects of oxidative stress and lipid peroxidation, the cell uses a number of protective mechanisms (Figure 10). The first line of defence against adduct formation is the prevention of oxidative stress. The body is able to protect itself against excessive ROS by neutralising them with various compounds, i.e., antioxidants. One such group is enzymatic antioxidants, such as superoxide dismutase (SOD), catalase (CAT) and glutathione peroxidase (GPx), and the other includes non-enzymatic antioxidants, such as vitamins C and E, carotenoids, flavonoids and glutathione (GSH) [63]. However, it is important to emphasise that an excess of vitamin C and E in the body can have a pro-oxidative effect [64,65]. Furthermore, a correlation was found between LA intake and adduct levels in rats fed a variety of vegetable oils. It was also observed that vitamin E content in sunflower oil did not significantly reduce the level of 1,*N*^2^-propano-2′-deoxyguanosine adducts [66], which resulted in their accumulation in the cell, thus hastening the ageing process and increasing the risk of mutations. This study shows that this one mechanism may not be very efficient.

Oxidative stress can also be relieved by reducing lipid peroxides to alcohols, which is performed by GPx together with GSH. This process also includes vitamin E, which as a lipophilic compound is able to interfere with lipid peroxidation [67]. Reactive 4-HNE is converted to an adduct with GSH by glutathione S-transferase (GTS) to prevent additional unwanted reactions [68]. It should be noted that studies found that the use of GSH biosynthesis inhibitors resulted in a fourfold increase in the level of 1,*N*^2^-propanodeoxyguanosine adducts in rat livers [69].

To provide genetic stability, living organisms have mechanisms of DNA repair. In the case of single-stranded DNA damage, the major mechanisms are base excision repair (BER) and nucleotide excision repair (NER). The BER pathway removes small base lesions that cause only little distortion to the DNA structure, whereas the NER pathway removes bulky lesions. In both mechanisms, the second undamaged strand is used as a matrix to provide damaged strand repair [70,71]. In the case of 4-HNE adducts, studies have shown that in human cells they are mainly repaired by the NER pathway. Interestingly, adducts **2** and **3** are found to be repaired more efficiently and rapidly than **1** and **4** [72].

For diseases whose etiology is based on faulty DNA repair machinery, such as Cockayne syndrome, Xeroderma pigmentosum (XP A-G), Trichothiodystrophy, or Fanconi anemia, those adducts are potentially harmful [73,74,75]. Xeroderma pigmentosum (XP) is one of the cancer-prone diseases characterized by a defective NER system. This mechanism plays a crucial role in the repair of ultraviolet-induced photoproducts in DNA, the major types being the cyclobutane pyrimidine dimer and the 6–4 photoproduct or its Dewar form [76]. An initial clinical diagnosis of XP can be made on the basis of either extreme sensitivity to UV or the appearance of lentigines on the face. XP may be connected with neurological abnormalities, including cognitive deterioration. Unfortunately, the cause of these neurological problems is not fully understood. XP patients have been assigned into eight complementation groups: A (XP-A) to complementation group G (XP-G), plus a variant type (XP-V). Mutations in seven of these genes (XP-A to XP-G) can result in XP and are associated with defects in nucleotide excision repair (NER). The products of these genes are involved in the repair of ultraviolet (UV)-induced damage in DNA. Defects in translesion synthesis carrying unrepaired DNA results in the accumulation of DNA lesions and may lead to neuronal death in XP patients [77]. It has also been proposed that the presence of such DNA damage accounts for DNA lesions that accumulate over time due to defective NER, leading to neuronal death in XP as a result of blocked transcription. One reason for this is that 1,*N*^2^-propano-2′-deoxyguanosine lesions could be removed from DNA strictly by the NER mechanism [72], and the repair pathway does not work properly in patients with XP [78]. Both DNA strands of active genes should be repaired effectively in neurons. The non-transcribed strand is needed as a template to repair the transcribed strand. A deficiency in this mechanism would be expected to result in neuronal death [79].

A similar situation connected with an NER system failure takes place in Cockayne syndrome [80] and trichothiodystrophy [81]. These disorders are connected with defects in NER mechanisms related to progressive neurological degeneration. These abnormalities may be caused by the faulty repair of DNA damage [82].

However, with efficient DNA repair mechanisms, proteins used in the BER or NER pathways may be modified by the formation of 4-HNE adducts which affect their activity. Experiments on BER pathway enzymes, OGG1, ANPG and TDG, have shown that 4-HNE form adducts with those enzymes, thus lowering their repair activity [83,84]. Inhibitory effects of 4-HNE on the NER repair pathway were also found, by studies on human cells with induced DNA damage by benzo[a]pyrene diol epoxide and UV [85].

## 6. Conclusions

ω-6 fatty acids are essential components of cell membranes and play important physiological roles. However, modern Western diets tend to contain excessive amounts of ω-6, which leads to an imbalance in the ω-6/ω-3 ratio. This imbalance has been associated with obesity, NAFLD, IBD and Alzheimer’s disease. Furthermore, this imbalance promotes chronic inflammation and alters membrane structure, resulting in oxidative stress and lipid peroxidation.

One of the primary byproducts of ω-6 lipid peroxidation is the reactive aldehyde-4-HNE, which readily forms adducts with DNA, compromising genomic stability. The NER pathway is primarily responsible for repairing DNA lesions induced by 4-HNE. However, this repair mechanism may be impaired in certain pathological conditions. Moreover, 4-HNE can also interact with proteins, modifying their structure and changing their function. Consequently, without a balanced diet, the body’s defence mechanisms may be insufficient.

Therefore, to mitigate the health risks associated with excessive ω-6 intake, it is crucial to improve the ω-6/ω-3 dietary ratio by increasing ω-3 intake and reducing consumption of processed foods rich in ω-6. Additionally, promoting a diet rich in antioxidants and whole foods may help to counteract oxidative stress and limit DNA damage. Public health strategies should focus on consumer education and encourage the reformulation of processed foods to reduce their ω-6 content.

Current evidence largely stems from in vitro and animal models, while human clinical studies remain limited. Future research should focus on evaluating the long-term health impacts of high ω-6 intake, defining safe intake thresholds for different populations, and clarifying the genotoxic and epigenetic effects of 4-HNE in human tissues. Understanding the relationship between dietary fat quality and molecular health outcomes is essential for developing dietary guidelines and promoting long-term disease prevention.

## Figures and Tables

**Table 2:** Biophysical properties of duplexes with different isomers [55,57,58].

5′-d(G-G-A-C-T-C-G-C-T-A-G-C)-3′ 3′-d(C-C-T-G-A-X-C-G-A-T-C-G)-5′			
X	T_m_ [°C]	Interstrand Cross-Link Formation *	Cross-Link Formation with Tetrapeptide *
dG	65	−	−
1	53	−	+
2	60	−	−
3	49	+	+
4	60	−	−

* ‘+’—cross-link formation observed; ‘−’—no formation.

## Data Availability

No new data were created or analyzed in this study. Data sharing is not applicable to this article.
